# Investigation of the first cluster of *Candida auris* cases among pediatric patients in the United States―Nevada, May 2022

**DOI:** 10.1017/ash.2023.400

**Published:** 2023-09-29

**Authors:** Sophie Jones, Kaitlin Forsberg, Christopher Preste, Joe Sexton, Paige Gable, Janet Glowicz, Heather Jones, Maroya Walters, Meghan Lyman, Chidinma Njoku, Kimisha Causey, Jeanne Ruff, Dallas Smith, Karen Wu, Elizabeth Misas, Teri Lynn, Chantal Lewis, Brian Min, Fathia Osman, Erin Archer

## Abstract

**Background:** *Candida auris* is a frequently drug-resistant yeast that can cause invasive disease and is easily transmitted in healthcare settings. Pediatric cases are rare in the United States, with <10 reported before 2022. In August 2021, the first *C. auris* case in Las Vegas was identified in an adult. By May 2022, 117 cases were identified across 16 healthcare facilities, including 3 pediatric cases at an acute-care hospital (ACH) with adult cases, representing the first pediatric cluster in the United States. The CDC and Nevada Division of Public and Behavioral Health (NVDPBH) sought to describe these cases and risk factors for *C. auris* acquisition. **Methods:** We defined a case as a patient’s first positive *C. auris* specimen. We reviewed medical records and infection prevention and control (IPC) practices. Environmental sampling was conducted on high-touch surfaces throughout affected adult and pediatric units. Isolate relatedness was assessed using whole-genome sequencing (WGS). **Results:** All 3 pediatric patients were born at the facility and had congenital heart defects. All were aged <6 months when they developed C. auris bloodstream infections; 2 developed C. auris endocarditis. One patient died. Patients overlapped in the pediatric cardiac intensive care unit; 2 did not leave between birth and *C. auris* infection. Mobile medical equipment was shared between adult and pediatric patients; lapses in cleaning and disinfection of shared mobile medical equipment and environmental surfaces were observed, presenting opportunities for transmission. Overall, 32 environmental samples were collected, and *C. auris* was isolated from 2 specimens from an adult unit without current cases. One was a composite sample from an adult patient’s bed handles, railings, tray table and call buttons, and the second was from an adult lift-assistance device. WGS of specimens from adult and pediatric cases and environmental isolates were in the same genetic cluster, with 2–10 single-nucleotide polymorphisms (SNPs) different, supporting within-hospital transmission. The pediatric cases varied by 0–3 SNPs; at least 2 were highly related. **Conclusions:** *C. auris* was likely introduced to the pediatric population from adults via inadequately cleaned and disinfected mobile medical equipment. We made recommendations to ensure adequate cleaning and disinfection and implement monitoring and audits. No pediatric cases have been identified since. This investigation demonstrates transmission can occur between unrelated units and populations and that robust infection prevention and control practices throughout the facility are critical for reducing *C. auris* environmental burden and limiting transmission, including to previously unaffected vulnerable populations, like children.

**Disclosures:** None

