# Investigation of the first cluster of Candida auris cases among pediatric patients in the United States―Nevada, May 2022 – CORRIGENDUM

**DOI:** 10.1017/ash.2025.60

**Published:** 2025-03-10

## ---

In the initial publication of this abstract, author Christopher Prestel’s surname was incorrect.^1^ The name has been corrected.
